# Predicting the Potential Distribution of *Aralia chinensis* L. (Wild Vegetable) in China Under Different Climate Change Scenarios

**DOI:** 10.3390/biology13110937

**Published:** 2024-11-16

**Authors:** Longjiang Liu, Shanshan Liang, Chengshi Xie, Jie Liu, Yaqiang Zheng, Juan Xue

**Affiliations:** College of Pharmacy, Guizhou University of Traditional Chinese Medicine, Guiyang 550025, China; liulongjiang106@gzy.edu.cn (L.L.); liangshanshan070@gzy.edu.cn (S.L.); xie177856@163.com (C.X.); liujie037@gzy.edu.cn (J.L.); zhengyaqiang131@gzy.edu.cn (Y.Z.)

**Keywords:** *Aralia chinensis* L., MaxEnt, potential distribution, suitable habitats

## Abstract

Global climate change has a main impact on the distribution of plants. *Aralia chinensis* L. is economically valuable, making it necessary to predict the impact of climate change on its distribution. It is important for researching the effects of climate change on *A. chinensis* distribution to achieve sustainable utilization. Based on 340 occurrence records of *A. chinensis* covering all known provinces and 58 environmental factor data, we used MaxEnt to simulate the potential distribution of *A. chinensis* under current and different future climate scenarios, analyzing the key environmental variables affecting its distribution. The results were as follows: (1) Suitable *A. chinensis* habitats under current and different future climate scenarios were mainly distributed in the southern region of China, east of the Hu Huanyong line. (2) Annual precipitation, minimum temperature during the coldest month, precipitation during the driest month, and slope were the key environmental variables affecting its potential distribution, and annual precipitation was more important. (3) Suitable habitat areas were projected to increase under different future climate scenarios and expand westward and northward while shrinking in the central regions, such as Hubei and Hunan. The results provide a theoretical reference for the conservation and cultivation of *A. chinensis*.

## Simple Summary

*Aralia chinensis* L. is an economic plant with high medicinal and food value, which is very popular in domestic and international markets. Global warming has an effect on the distribution of a large number of species; accordingly, it is necessary to study the effects on climate change on *A. chinensis* in order to protect and utilize *A. chinensis* resources much better. The maximum entropy model (MaxEnt) was used to predict the distribution of *A. chinensis* under the influence of climate change. It was found that *A. chinensisis* mainly distributed in the southern of China, east of the Hu Huanyong line under current and different future climate scenarios, and its distribution range might be expanded to the north and west in the future period; on the contrary, the middle part of its distribution range might be contracted. Precipitation had a great influence on the distribution, especially annual precipitation. It is recommended to protect *A. chinensis* resources in the central part of its distribution range in the future, and to collect *A. chinensis* resources in the region, and this work can help to customize relevant control strategies.

## 1. Introduction

Rapid urban development since the Industrial Revolution has resulted in high greenhouse gas emissions [1]. Compared with the period from 1995 to 2014, global temperatures could increase by 0.7–4.0 °C by approximately 2100 [2]. Climate change is therefore a major challenge to the survival of many species. For example, the number of tree species at risk of extinction is high, mainly due to climate change and the loss of habitat caused by human activities [3].

*Aralia chinensis* is a traditional Chinese mountain wild vegetable commonly used in both food and Chinese medicine. It has a rich, wild flavor, sweet and mellow taste, and is known as “the king of wild vegetables”, highly favored by consumers. It has analgesic and anti-inflammatory effects, dispels wind, promotes qi, dispels dampness, and promotes blood circulation. The root bark can be used to treat gastritis, nephritis, and rheumatic pain, and it can be applied externally to knife wounds [4]. The young stems and buds of *A. chinensis* that sprout in the spring are the main edible plant parts. It is one of the main wild vegetables exported for commerce [5]. *A. chinensis* is found in forests, thickets, or on forest edge roadsides. It is widely distributed in China: north from southern Gansu, southern Shaanxi, southwestern Shanxi, central Hebei; south to northwestern and central Yunnan, northwestern and northeastern Guangxi, northern Guangdong, southwestern Fujian, and eastern Fujian; west from northwestern Yunnan; and east to the coast. It has a vertical distribution from the coast to an altitude of 2700 m [4]. Owing to the annual decrease in natural forest areas and tree planting on the slopes of barren mountains, coupled with destructive and predatory harvesting in some areas and cut stem transplanting, the remaining *A. chinensis* area is decreasing annually and is threatened with extinction in some areas [5]. In 2004, *A. chinensis* was listed as vulnerable on the IUCN Red List of Threatened Species (IUCN 2004 ver. 3.1)—Vulnerable (VU).

Owing to its economic value, *A. chinensis* has been widely introduced and artificially cultivated in recent years [6,7,8]. However, there are problems associated with its introduction and cultivation. The successful introduction and cultivation of *A. chinensis* requires an understanding of the environmental conditions that affect its growth to select the most suitable habitats [9]. Determining how *A. chinensis* will adapt to global climate change is also critical. Important changes in climatic conditions have occurred globally, and the changing climatic environment has impacted plant distributions [10].

Species distribution models are popular analytical tools used in ecology, predicting and managing the impact of climate change on species distribution [11,12]. Current species distribution models include the Bioclimatic Analysis and Prediction System (BIOCLIM) [13], Genetic Algorithm for Rule Set Generation (GARP) [14], Ecological Niche Factor Analysis (ENFA) [15], and maximum entropy (MaxEnt) [16,17]. Among these modeling approaches, MaxEnt is a high-performance method [18], used to select a probability distribution to maximize entropy under known constraints [19]. It is widely used for estimating the potential growth ranges of species [20,21].

To date, previous studies on *A. chinensis* have mainly focused on its chemical constituents [22] and pharmacology [23], but no studies have been conducted to predict its distribution. Therefore, in this study, the potential distribution of *A. chinensis* was predicted to (1) the places where the current distribution of *A. chinensis* are; (2) the key environmental factors that affect the distribution of *A. chinensis*; and (3) the distribution trend of *A. chinensis* in the future period. The findings provide scientific guidance for strategic site selection for *A. chinensis* in China and associated resource conservation and cultivation.

## 2. Materials and Methods

### 2.1. Distribution Data Collection and Processing

**Figure 1:**
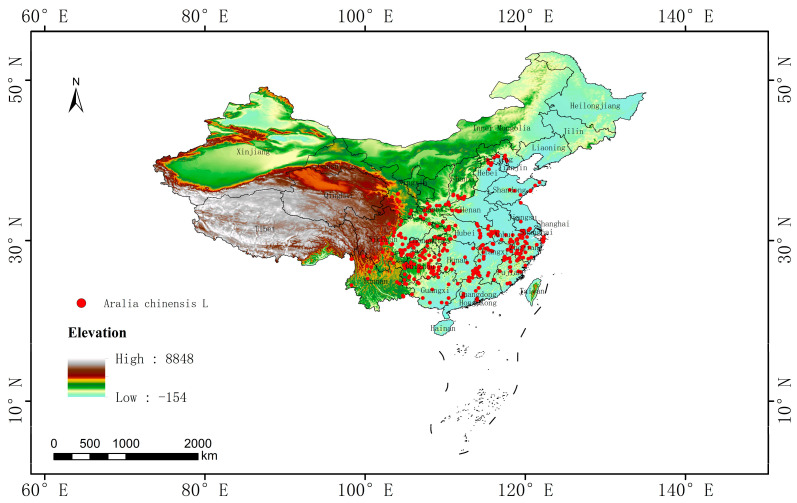
Spatial distribution of *Aralia chinensis* L. in China.

Of the data from the National Specimen Information Infrastructure (http://www.nsii.org.cn/, accessed on 21 November 2023), Chinese Virtual Herbarium (https://www.cvh.ac.cn/, accessed on 23 November 2023), and Plant Photo Bank of China (http://ppbc.iplant.cn/, accessed on 25 November 2023), 369 records were obtained by eliminating incorrect and repeated distribution data. As the data were collected from multiple platforms, they were often highly similar. ArcGIS 10.8 was used to ensure that the distance between any two occurrence records was >5 km [24]. A total of 340 *A. chinensis* distribution data points covering all known provinces were finally obtained for modeling (Figure 1).

### 2.2. Acquisition and Environmental Factor Data Processing

Fifty-eight environmental factors that may influence the distribution of *A. chinensis* were examined, including 19 bioclimatic, 3 topographic, and 36 soil factors (Table S1). The bioclimatic factors were obtained from the WorldClim website ver. 2.1 (https://www.worldclim.org/, accessed on 3 December 2023), which include baseline climate data (current (average from 1970 to 2000)) and future climate data under two scenarios (SSP126 and SSP585) (2030S (average from 2021 and 2040), 2050S (average of 2041–2060), 2070S (average from 2061 to 2080), and 2090S (average from 2081 to 2100)). Of these, SSP126 uses a sustainable development path representing low greenhouse gas (GHG) emissions, and SSP585 represents high levels of greenhouse gas emissions using a fossil fuel-based development approach [25,26]. ArcGIS was used to process the topographic data obtained from the WorldClim website ver. 2.1 (https://www.worldclim.org/, accessed on 4 December 2023) to obtain elevation, aspect, and slope data. Soil variable data were obtained from the Harmonized World Soil Database ver. 1.2 (https://www.fao.org/, accessed on 7 December 2023).

To avoid model overfitting, 58 environmental factors were introduced into the model and run repeatedly 10 times using default parameters [27]. Variables with a contribution of 0 were then removed using jackknife tests. A correlation analysis was performed on the environmental factor data extracted by ArcGIS. If the correlation coefficient (R) was ≥0.8, the environmental variables with a large contribution rate and notable biological significance were retained [28,29]. A total of 14 environmental factors were retained for modeling (Table 1).

### 2.3. MaxEnt Model Construction and Evaluation

The coordinates of the 340 *A. chinensis* distribution records were converted to the American Standard Code for Information Interchange (ASCII) format using the ArcGIS 10.8 software and then imported to MaxEnt. The test set comprised 25% of distribution records and the remaining data were used as a training set. The other settings used were default values, and the regularization multiplier (RM) was set to 1. The output simulation results were presented in Logistic format and ASCII files; runs were repeated 10 times. The size of the area under the curve (AUC) was used to measure the accuracy of the model’s predictions, with values in the range of [0,1]; a model is considered highly accurate if the AUC is > 0.9 [30]. The importance and contribution rates of the environmental variables were obtained through the jackknife method; their values related to the degree of influence on the distribution of the species. The response curve of environmental factors reflects the relationship between environmental factors and the probability of species existence [31]. The size of the environmental factors corresponding to the probability of existence > 0.5 is more conducive to the growth of the species [32].

### 2.4. Classification of Suitable Habitats

The natural breakpoint grading method was used to classify suitable areas into four classes using the ArcGIS 10.8 software [33]: non-suitable habitats (0–0.3); relatively unsuitable habitats (0.3–0.5); moderately suitable habitats (0.5–0.8); and highly suitable habitats (0.8–1). We calculated the actual area of each suitable habitat in the future and analyzed the trends for suitable habitats accordingly.

## 3. Results

### 3.1. Evaluation of Model Prediction Accuracy

As shown in Table 2, the AUC values all exceeded 0.9 under current and different future climate scenarios: the larger the AUC, the stronger the judgment of the model. Ideally, the model’s predicted distribution area and the actual distribution area of the species should match exactly, and the AUC value was 1 in this case [30]. The results for MaxEnt were credible and provided good prediction accuracy to simulate the distribution of A. chinensis [34].

### 3.2. Projections of Potential Suitable Distribution Areas Under Current Climate Scenarios

**Figure 2:**
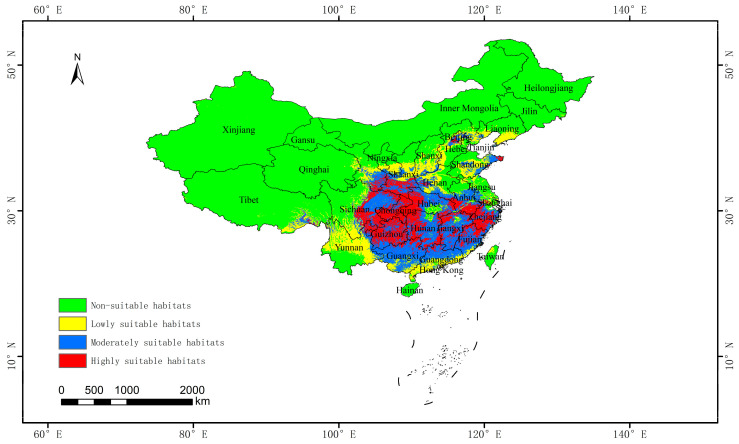
Distribution of suitable habitats for *Aralia chinensis* L. in China under current climate scenarios.

Figure 2 showed that there were 22 suitable habitats which included the most areas within Chongqing, Guizhou, Hunan, Hubei, Guangdong, Guangxi, Jiangxi, Fujian, Zhejiang, and Beijing, and in some areas of Gansu, Ningxia, Shaanxi, Shanxi, Hebei, Henan, Shandong, Jiangsu, Anhui, Yunnan, Sichuan, and Tibet. Ten highly suitable habitats included Guizhou, Chongqing, Sichuan, Hubei, Shaanxi, Jiangxi, Anhui, Fujian, Zhejiang, and Hunan. Nine moderately suitable habitats included Guangdong, Guangxi, Fujian, Jiangxi, Hunan, Hubei, Anhui, Sichuan, Gansu, and other areas. The distribution range of *A. chinensis* is within the southern region of China, east of the Hu Huanyong line.

Areas that provide suitable habitats under the current climate scenario were extracted using ArcGIS 10.8 (Table S2). Table S2 indicates that the current total suitable habitat for *A. chinensis* was 269.86 × 10^4^ km^2^, accounting for 28.07% of the national territory, and the non-suitable habitats covered 691.43 × 10^4^ km^2^. Moderately and highly suitable habitats covered 109.28 × 10^4^ and 73.57 × 10^4^ km^2^, accounting for 11.37 and 7.65% of the national territory, respectively. Guizhou Province provided the largest highly suitable habitat of 10.66 × 10^4^ km^2^; Sichuan Province provided the largest suitable habitat of 26.59 × 10^4^ km^2^; and Sichuan Province provided the largest moderately suitable habitat of 15.35 × 10^4^ km^2^. The largest proportion of total suitable habitats was within Guizhou Province (99.98%), comprising the largest proportion of highly suitable habitats (99.86%).

### 3.3. Simulation and Change Analysis of Suitable Habitats for A. chinensis Under Future Climate Change Scenarios

**Figure 3:**
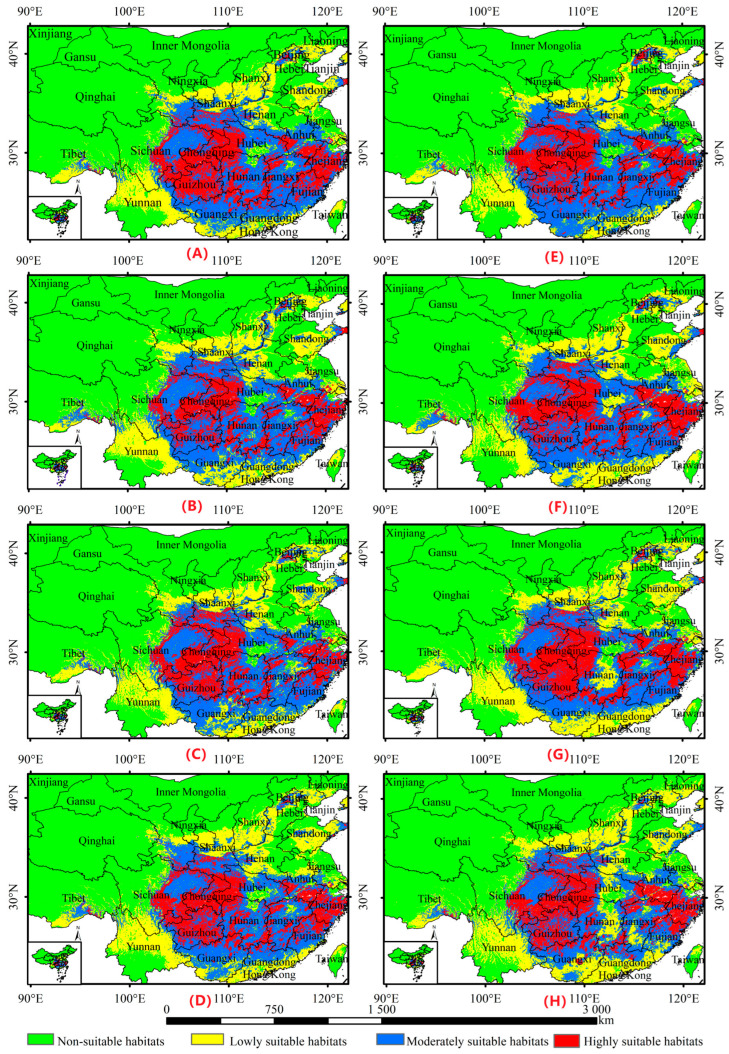
Distribution of suitable habitats for *Aralia chinensis* L in China under different future climate scenarios, including (**A**) SSP126-2030S, (**B**) SSP126-2050S, (**C**) SSP126-2070S, (**D**) SSP126-2090S, (**E**) SSP585-2030S, (**F**) SSP585-2050S, (**G**) SSP585-2070S, and (**H**) SSP585-2090S.

**Figure 4:**
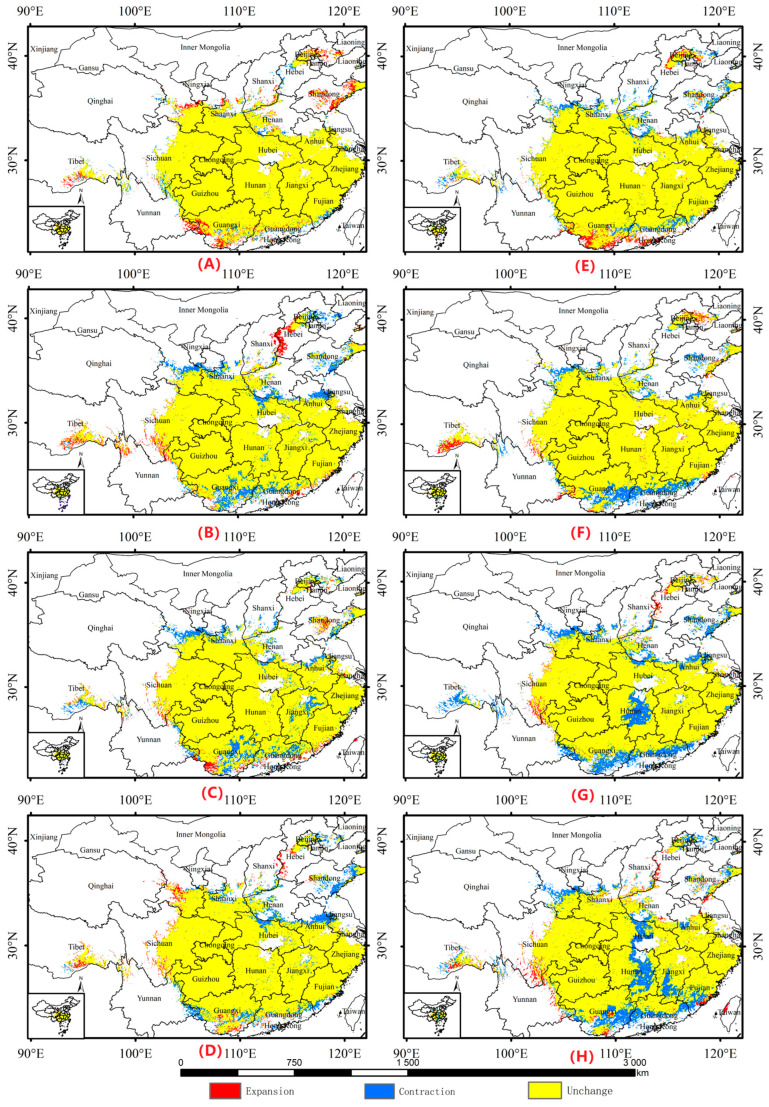
Unchanged, contracted, and expanded suitable habitats for *Aralia chinensis* L. in China under different future climate scenarios, including (**A**) SSP126-2030S, (**B**) SSP126-2050S, (**C**) SSP126-2070S, (**D**) SSP126-2090S, (**E**) SSP585-2030S, (**F**) SSP585-2050S, (**G**) SSP585-2070S, and (**H**) SSP585-2090S.

Figure 3 shows that the distribution of *A. chinensis* under different future climate scenarios was generally consistent with that of the current period. However, the area of total suitable habitats increased, and the areas of increase were mainly within Hebei, Sichuan, and Tibet. The area of highly suitable habitat decreased, and the areas of decrease were mainly distributed in Hunan, Hubei, Shaanxi, Guizhou, Gansu, and Jiangxi. The area of moderately suitable habitats first increased and then decreased; the areas of increase were within Shaanxi, Guizhou, and Taiwan, and the areas of decrease were in Guangxi, Guangdong, and Zhejiang. The area of relatively unsuitable habitats increased mainly in Hunan, Guangxi, and Guangdong (Table S3). Suitable habitats will expand to the southern part of Gansu Province and west to the central part of Sichuan Province. The northern part may expand to some areas of Hebei and Shandong. In the south, Guangxi and Guangdong contracted northward and shrank in central regions, such as Hubei and Hunan (Figure 4).

### 3.4. Predicting Suitable Habitat Center Migration Trajectories Under Different Climate Scenarios

**Figure 5:**
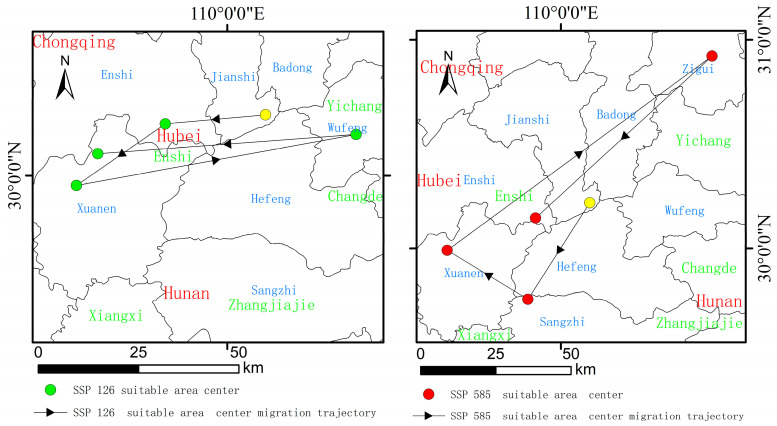
Suitable habitat center migration trajectory under the SSP126 and SSP585 climate scenarios.

Figure 5 shows that the center of the current suitable habitat for *A. chinensis* is located in Jianshi County, Enshi Prefecture, Hubei Province (110.14° E, 30.22° N). Under the SSP126 scenario, the center of the suitable habitat of *A. chinensis* would shift westward to Enshi County, Enshi Prefecture, Hubei Province (109.78° E, 30.19° N) in the 2030s, southwestward to Xuan’en County, Enshi Prefecture, Hubei Province (109.46° E, 29.96° N) in the 2050s, and eastward to Wufeng County, Yichang City, Hubei Province (110.47° E, 30.15° N) in the 2070s. In the 2090s, it would shift southwestward to Xuan’en County, Enshi Prefecture, Hubei Province (109.53° E, 30.08 °N). Under the SSP585 scenario, the center of the suitable habitat for *A. chinensis* would shift southwestward to Sangzhi County, Zhangjiajie City, Hunan Province (109.84° E, 29.76° N) in the 2030s, northwestward to Xuanen County, Enshi Prefecture, Hubei Province (109.45° E, 29.99° N) in the 2050s, and northeastward to Zigui County, Yichang City, Hubei Province (110.73° E, 30.92° N) in the 2070s. In the 2090s, it would shift southwest to Enshi City, Enshi Prefecture, Hubei Province (109.88° E, 30.15° N).

### 3.5. Key Environmental Factors Affecting A. chinensis Distribution

**Figure 6:**
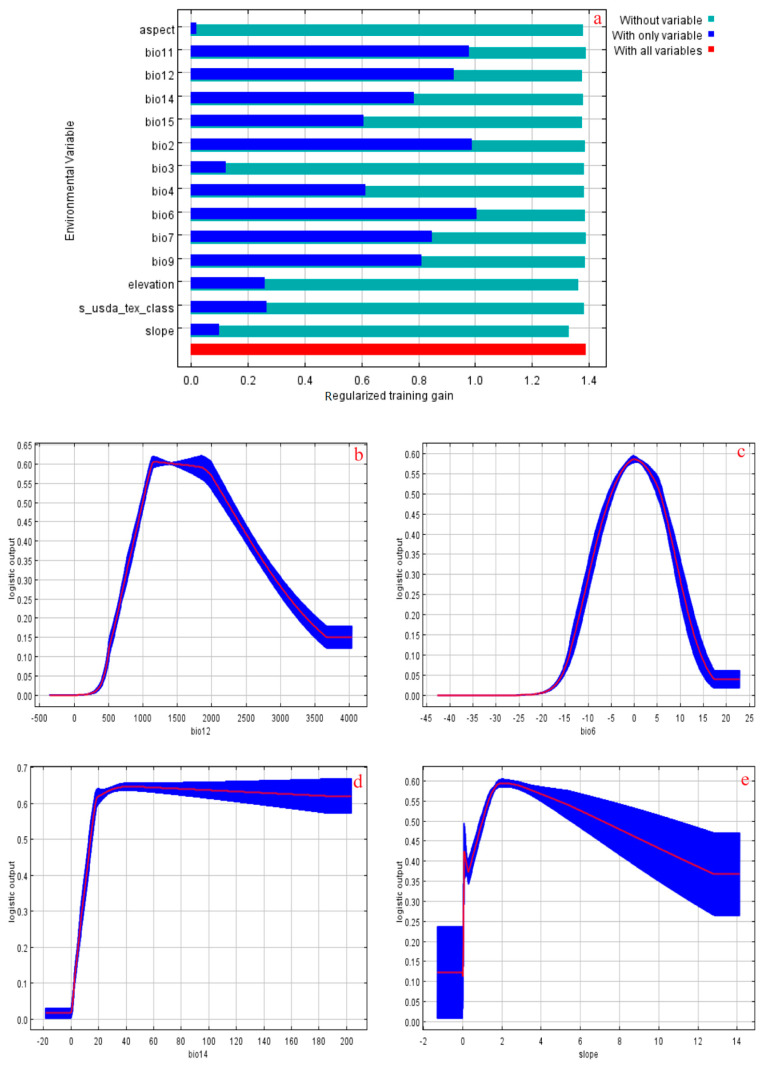
(**a**) Evaluation of the major environmental factors using the jackknife method, and (**b**–**e**) response curves for the dominant environmental variables. The probability values shown are the average over 10 replicate runs. The blue margins show the standard deviation (±SD) calculated for 10 replicates.

Table 3 indicates that bio12, bio6, bio14, slope, and bio4 were the variables with the greatest contributions, providing a cumulative contribution of 84.1% to the model predictions. The knife-cut test of regularization training gain showed that bio6 was the most important factor; it affected the potential distribution of *A. chinensis* when used alone. When predicting the distribution of *A. chinensis* omitting slopes, the impact was greater (Figure 6a). Taken together, bio12, bio6, bio14, and slope were the key environmental variables affecting the potential distribution of *A. chinensis*, and annual precipitation was much more important, with a percentage contribution of 36.6.

The response curves between the occurrence probability of *A. chinensis* and the main environmental factors were obtained using the MaxEnt model (Figure 6b–e). Figure 6b–e show that the response curves of bio12 and bio6 followed a normal distribution. With an increase in bio12, the likelihood of *A. chinensis* existing increased rapidly, reaching a maximum when it was close to 2000 mm. Subsequently, the probability of *A. chinensis* existing decreased rapidly with an increase in bio12. Similarly, when bio6 was near −20 °C, the probability of existence was near 0, and it then increased rapidly. When bio6 was near 2 °C, the probability of existence reached its maximum and it then decreased rapidly. The bio14 response curve shows that, with an increase in bio14, the probability of *A. chinensis* existing increased rapidly, but when it reached approximately 20 mm, the probability of *A. chinensis* existing remained unchanged. The response curve for slope shows that the likelihood of *A. chinensis* existing rapidly increased with an increase in slope, reaching a maximum when it was near 2°. Subsequently, the likelihood of *A. chinensis* existing gradually decreased with an increase in slope.

As listed in Table 4, bio12 in suitable *A. chinensis* habitats ranged from 725.65 to 3,024.54 mm, and the optimum amount of precipitation was 1,989.16 mm. The optimum values for bio6, bio14, and slope were 1.73 °C, 18.80 mm, and 2.07–2.78°, respectively.

## 4. Discussion

### 4.1. Accuracy of A. chinensis Distribution Prediction

MaxEnt is widely used to examine the distribution of various plants, insects, and fungi [35]. To simulate species distribution, using a small amount of species sample data is effective. However, the size of the sample had a direct impact on the accuracy and stability of the model in predicting the distribution of species. When sufficient species distribution points are used to construct a model, its transferability is guaranteed [36,37]. In this study, a total of 340 sample points covered all known provinces. The prediction results of this study showed that *A. chinensis* was mainly distributed in most areas of Chongqing, Guizhou, Hunan, Hubei, Guangdong, Guangxi, Jiangxi, Fujian, Zhejiang, and Beijing, and in parts of Gansu, Ningxia, Shaanxi, Shanxi, Hebei, Henan, Shandong, Jiangsu, Anhui, Yunnan, Sichuan, and Tibet. These results are basically consistent with the distribution range of *A. chinensis* recorded in the *Flora of China* [4].

### 4.2. Environmental Factors Affecting Distribution of A. chinensis

Climate is a major factor influencing the distribution of species over large scales in geographic regions [38]. Topography is important for species distribution at small scales [39,40]. Bio12, bio6, bio14, and slope were the key environmental variables that affected the potential geographical distribution of A. chinensis. Two factors, bio12 and bio14, were related to precipitation, and their accumulated contribution rate reached 54.8%. Precipitation can limit and affect species distribution in a variety of ways [41]. For example, excessive water can cause root rot. Aralia chinensis prefers a humid air environment, but its roots are not resistant to flooding. If water accumulates for three days, it can lead to widespread death; high soil moisture can cause root rot [42,43]. After August, A. chinensis is susceptible to drought and it gradually stops growing, which reduces its water demand. Our study predicted that the distribution range of A. chinensis is in the southern region of China, east of the Hu Huanyong line.

The 400 mm isohyet precipitation line discovered in modern times is the boundary between the semi-humid and semi-arid areas in China, which coincides with the Hu Huanyong line. The prediction results showed that, in suitable *A. chinensis* habitats, bio12 ranged from 725.65 to 3024.54 mm; its optimal value was approximately 1989.16 mm. Furthermore, bio14 ranged from 7.70 to 203.50 mm; its optimal value was approximately 18.80 mm. Temperature also affects plant distribution, especially for tropical and subtropical species. Low temperatures are often the main factor limiting their northward distribution [44]. Lower temperatures in the coldest months lead to frost damage and even plant death [41]. In suitable *A. chinensis* habitats, bio6 ranged from –10.98 to −28 °C; the optimum value was approximately 1.73 °C. Omitting slope had a substantial influence on the distribution of *A. chinensis*. This plant species prefers fertile, permeable, and well-drained loam. Our study showed that, in suitable *A. chinensis* habitats, the slope ranged from 0.07 to 10.81°; its optimal value was 2.07–2.78°.

### 4.3. Effects of Climate Change on the Distribution of A. chinensis

Under different future climate scenarios, suitable habitats for *A. chinensis* will expand westward to central Sichuan Province and southern Gansu Province. The northern part may expand to parts of Hebei and Shandong. In the south, Guangxi and Guangdong may shrink northward, as well as in central regions, such as Hubei and Hunan. Warming affects the distribution pattern of temperature and precipitation in different regions [45]. According to the China Climate Change Blue Book (2024) [46], the global average temperature over the last 10 years was approximately 1.2 °C higher than the pre-industrial level. The average annual precipitation in China increased by an average of 5.2 mm per decade from 1961 to 2023. With global warming, China’s rainfall belt has gradually moved northward [47]. Recently, the variation in flood season precipitation in the Yangtze River Basin has increased significantly, especially since the 1990s, when several major floods occurred [48]. Approximately 7500–5000 years ago, when the temperature rose after the end of the Dali Ice Age, the temperature was 2–3 °C higher than that at present, according to plant pollen analyses. Precipitation amounts were also 500–600 mm higher than those at present. At that time, there were several large lakes in the Yangtze River Basin, including the Yun-meng and Peng-li lakes [49]. The Yun-meng lakes were a group of ancient lakes on the Jianghan Plain, which had a circumference of approximately 450 km and included today’s southern Hubei and northern Hunan Provinces. Global climate change has led to various climatic and environmental problems. This has consequently affected the growth and development of trees through physiological stresses, such as temperature and precipitation [50]. Environmental problems, such as rising temperatures caused by global warming, the northward shift in the rainfall zone in China, the increase in precipitation in China, and great floods in the Yangtze River Basin, may affect the growth and development of *A. chinensis* through the physiological stresses of temperature and precipitation, which may have an impact on the distribution of *A. chinensis* in future suitable habitats. Artificial reservoirs do change climate patterns in arid regions, especially in terms of localized precipitation and daily temperature changes, with higher relative humidity in riparian forested areas [51]. Waterlogging can lead to the death of *A. chinensis*. Further, does the contraction of Hunan and Hubei in the future fitness zone mean the return of the Yun-meng lakes?

### 4.4. Significance and Limitations of the Study

*A. chinensis* L. has considerable economic value. Research on climate change is of great practical significance for *A. chinensis* conservation management and its sustainable development. In the coming period, most of the existing areas are suitable for the distribution of it. Therefore, it is recommended that the wild natural populations of this species should be locally protected and the *A. chinensis* industry can continue to be developed in these areas. However, climate change will result in the extinction of this species in some areas in the future, which will affect the sustainable utilization of *A. chinensis*, and the germplasm of *A. chinensis* should be collected early for conservation. These potential extinction sites require attention. Bio12, bio6, bio14, and slope affect the distribution of *A. chinensis*, which has implications for its cultivation. In this study, the effects of climate, soil, and topography were considered when predicting the distribution of *A. chinensis*. However, there are still limitations to our estimations, such as whether using the environmental data such as human impacts, vegetation cover, soil quality, and mid-decade climate factors can improve prediction accuracy. However, although human impact factors affect the distribution of this species [5], because the reliable human impact factors in the future were not available [45], human impact factors were not considered in this prediction study. Additionally, the MaxEnt model is currently considered an ideal predictive tool compared with other species distribution models, but cross-validation with other models enhances the reliability.

## 5. Conclusions

Our modeling predictions suggest that suitable habitats for *A. chinensis* under current and different future climate scenarios are within the southern part of China on the east side of the Hu Huanyong line. The total suitable area under different future climate scenarios is predicted to increase, and areas will expand westward and northward, but will shrink in central regions, such as Hubei and Hunan. Bio12, bio6, bio14, and slope were the key environmental variables affecting the potential distribution of *A. chinensis*. These results provide a theoretical reference for the cultivation and sustainable use of *A. chinensis*.

## Figures and Tables

**Table 1:** Environmental variables used to predict the distribution of *Aralia chinensis* L.

Variable	Description	Percent Contribution	Permutation Importance
Bio2	Mean diurnal range	2.3	11
Bio3	Isothermality	2.1	1.2
Bio4	Temperature seasonality	5.1	7.5
Bio6	Minimum temperature of the coldest month	17.3	5.7
Bio7	Temperature annual range	1.6	10.4
Bio9	Mean temperature of driest quarter	1.4	1.4
Bio11	Mean temperature of coldest quarter	1.5	0.9
Bio12	Annual precipitation	34.4	6.6
Bio14	Precipitation of the driest month	11.8	3.1
Bio15	Precipitation seasonality	1.3	2.5
Aspect	Aspect position of species	1.2	2
Elevation	Elevation position of species	2.4	5.5
Slope	Slope position of species	6.1	7.7
S_usda_tex_class	Subsoil USDA texture classification	1	0.3

**Table 2:** AUC values of simulation results.

Climate Scenario and Year	AUC Value	Random Error
Current	0.919	0.003
SSP126-2030S	0.920	0.005
SSP126-2050S	0.925	0.004
SSP126-2070S	0.925	0.005
SSP126-2090S	0.920	0.004
SSP585-2030S	0.922	0.003
SSP585-2050S	0.919	0.003
SSP585-2070S	0.925	0.003
SSP585-2090S	0.923	0.004

**Table 3:** Importance of each dominant environmental variable in the MaxEnt model.

Variable	Percentage Contribution	Permutation Importance
Bio12	36.6	23.7
Bio6	19.1	4.3
Bio14	18.2	3.9
Slope	5.4	4.4
Bio4	4.8	5.4
Elevation	3.6	13.4
Bio3	2.7	1.2
Bio7	2.2	19
Bio2	1.7	11.6
Bio15	1.5	4
S_usda_tex_class	1.2	0.7
Bio9	1.2	3.7
Bio11	1	3.2
Aspect	0.8	1.5

**Table 4:** Range of dominant environmental variables in suitable habitats for *Aralia chinensis* L.

Environmental Variables	Range	Optimal Value	Unit
Bio12	725.65–3024.54	1989.16	mm
Bio6	−10.98 to 8.28	1.73	℃
Bio14	7.70–203.50	18.80	mm
Slope	0.07–10.81	2.07–2.78	°

## Data Availability

The data are included in the article. For the data provided in this study, see Section 2.1 and Section 2.2 in the text.

## Supplementary Materials

The following supporting information can be downloaded from: https://www.mdpi.com/article/10.3390/biology13110937/s1, Table S1: Fifty-eight environment variables; Table S2: The suitable habitat areas for *Aralia chinensis* L in different provinces under current climate scenarios; Table S3: The suitable habitat areas for *Aralia chinensis* L in different provinces under different future climate scenarios.
